# EMG-based pattern recognition approach in post stroke robot-aided rehabilitation: a feasibility study

**DOI:** 10.1186/1743-0003-10-75

**Published:** 2013-07-15

**Authors:** Benedetta Cesqui, Peppino Tropea, Silvestro Micera, Hermano Igo Krebs

**Affiliations:** 1 Laboratory of Neuromotor Physiology, Santa Lucia Foundation, via Ardeatina 306, 00179, Rome, Italy; 2 BioRobotics Institute, Scuola Superiore Sant’Anna, Pisa, Italy; 3 Translational Neural Engineering Lab, Center for Neuroprosthetics and Institute of Bioengineering, Ecole Polytechnique Federale de Lausanne, Lausanne, Switzerland; 4 Newman Laboratory for Biomechanics and Human Rehabilitation, Department of Mechanical Engineering, MIT Massachusetts Institute of Technology, 77 Massachusetts Avenue., Cambridge, MA, 02139, USA; 5 Department of Neurology and Division of Rehabilitative Medicine, University of Maryland School of Medicine, Baltimore, MD, USA

## Abstract

**Background:**

Several studies investigating the use of electromyographic (EMG) signals in robot-based stroke neuro-rehabilitation to enhance functional recovery. Here we explored whether a classical EMG-based patterns recognition approach could be employed to predict patients’ intentions while attempting to generate goal-directed movements in the horizontal plane.

**Methods:**

Nine right-handed healthy subjects and seven right-handed stroke survivors performed reaching movements in the horizontal plane. EMG signals were recorded and used to identify the intended motion direction of the subjects. To this aim, a standard pattern recognition algorithm (i.e., Support Vector Machine, SVM) was used. Different tests were carried out to understand the role of the inter- and intra-subjects’ variability in affecting classifier accuracy. Abnormal muscular spatial patterns generating misclassification were evaluated by means of an assessment index calculated from the results achieved with the PCA, i.e., the so-called Coefficient of Expressiveness (CoE).

**Results:**

Processing the EMG signals of the healthy subjects, in most of the cases we were able to build a static functional map of the EMG activation patterns for point-to-point reaching movements on the horizontal plane. On the contrary, when processing the EMG signals of the pathological subjects a good classification was not possible. In particular, patients’ aimed movement direction was not predictable with sufficient accuracy either when using the general map extracted from data of normal subjects and when tuning the classifier on the EMG signals recorded from each patient.

**Conclusions:**

The experimental findings herein reported show that the use of EMG patterns recognition approach might not be practical to decode movement intention in subjects with neurological injury such as stroke. Rather than estimate motion from EMGs, future scenarios should encourage the utilization of these signals to detect and interpret the normal and abnormal muscle patterns and provide feedback on their correct recruitment.

## Background

The American Heart Association, the Department of Veterans Affairs and the Department of Defense have recently endorsed the use of robotic therapy to enhance the recovery of upper extremity following a stroke [1]. This endorsement is a result of multiple, randomized controlled clinical studies that showed improvement of movement coordination and motor recovery after injury [2-6]. The key concept behind upper extremity robotic therapy is that robot-based training involving repetitive task-oriented movements with significant attention demands might promote brain plasticity and recovery. While motor recovery has several distinct traits other than motor learning, we often adopt motor learning models as the keystone for organizing therapy aimed at altering the underlying neural architecture and connectivity to promote recovery. Yet little is known about what constitutes best practice after a stroke. We do not know the optimal amount of therapy, the most effective duration of treatment, its content, and the best intensity of the training sessions [7-10]. Nevertheless, the potential benefits of robotic therapy are considerable. For example, a “robotic gym” could allow the rehabilitation of several patients to take place at the same time with only one therapist present or to modify the kind of robot and its specific use during the different phases of the recovery of the patient. The therapist role could evolve into a supervisory function in which s/he selects from among different types of protocols the optimal one for the particular patient. Interesting information that might facilitate the therapist’s choice from the repertoire of possibilities to optimize motor recovery can be gathered from electromyographic (EMG) signals [9,11,12]. EMG signals could allow us to predict in advance the desired motor task - as these signals always start several milliseconds prior to an action initiation - but this possibility could be highly influenced by specific deficits. Several methods have been developed in order to estimate different features of the movements from muscle activity, i.e. joint angles, velocity torques, and stiffness [13-19]. In particular, many groups suggested that muscle synergies (coordinated recruitment of groups of muscles with specific activation profile) could be the basic control modules on which the CNS relies to generate motion since the dynamic behavior of the musculoskeletal system seems to be captured by the structure of the synergies [20-23]. Moreover, it has been suggested that the assessment of muscle synergies should be used to evaluate different therapy modalities in post-stroke rehabilitation [24]. These results support the idea that we might employ EMG signals to enhance robotic therapy.

While the use of EMG signals for biofeedback in rehabilitation has been quite controversial [25-27], few attempts have been made to integrate them with robotic therapy so far. To our knowledge, the only systematic application of EMG has been employed in the e-100 Neurorobotic System from Myomo (Myomo, Inc., Boston, MA, USA) with a non-invasive EMG platform, able to use the signals to understand whether the subjects self-initiated and controlled movement and to provide help if necessary. To date, only small uncontrolled studies have been performed with the system [28]. Hu and colleagues [29] also compared outcomes of a group receiving robotic wrist therapy assisted by an EMG-driven algorithm with a group receiving passive wrist motion, demonstrating better outcomes for the intention-driven group. These results indicate the potential for employing EMG signals to enhance robotic therapy.

Here we investigated whether EMG signals could correlate the activations of the upper arm muscle patterns with goal-directed movements, and whether this kind of information can be used for movement prediction. Our goal was to extract a general model/map of the EMG activation patterns valid for point-to-point reaching movements in a gravity-compensated, horizontal plane environment. This information could potentially be used in future applications to develop an assistive robotic device able to discriminate between the intended (i.e., determined by the specific task) and the generated (i.e., based on the recorded muscle activity) movement directions, and eventually to provide assistance when the two are not coincident. In this context different scenarios are possible. In fact, the direction error could be detected by either comparing the real-time EMG signals to a reference model of patterns of activation extracted from the healthy population, or by allowing the patient to establish his own target movements by calibrating the system for every pathological individual.

To achieve our goal, young healthy and pathological subjects were asked to perform pointing movements on the horizontal plane while holding the handle of a robotic manipulandum. The EMG signals of several muscles of the arm and of the trunk were recorded. We applied a classical electromyography pattern classification technique used for similar applications [30]. In particular, a standard pattern recognition method known as Support Vector Machine was used to identify the intended movement direction from the muscle activities relative to the first instants of motion. Moreover, an analysis of EMG spatial characteristics was carried out to detect anomalies and spurious coactivational patterns. The aim of our analysis was to understand how the inter- and intra-subjects’ variability affected the repeatability of the measurements, hence the classifier accuracy, both in the case of healthy subjects and post stroke patients.

## Methods

### Subjects

Nine right-handed young healthy subjects (GROUP I) and seven stroke survivors (GROUP II) of different impairment levels volunteered to participate in the experiment. The following inclusion criteria were applied for patient recruitment: 1) diagnosis of a single, unilateral stroke verified by brain imaging; 2) sufficient cognitive and language abilities to understand and follow instructions; 3) absence of apraxia and severe concurrent medical problems (including shoulder pain). Table 1 summarizes the features and the clinical assessment of all patients, ordered with respect to the impairment level on the base of the Fugl-Meyer clinical rate for loss of sensorimotor function in the arm [31]. Patients #2 and #6 were out-patients of the Burke Rehabilitation Hospital (White Plains, NY). The other patients were inpatients (i.e., Patient #4, #5, and #7) and outpatients (i.e., Patient #1, #3) of the Neurological and Severe Brain Injury Unit of Auxilium Vitae Rehabilitation Center (Volterra, Italy). The experiments were carried out during the first therapy day of the rehabilitation program. Experiments with healthy subjects were carried out at the Department of Mechanical Engineering of the Massachusetts Institute of Technology, Cambridge, MA. The experimental protocol was approved by the Committee on the Use of Human Experimental Subjects of the Massachusetts Institute of Technology (COUHES) and the institutional review boards of Burke Rehabilitation Hospital and the Auxilium Vitae Rehabilitation Center. All subjects gave their informed consent before the experimental session.

**Table 1:** Demographic and clinical data of patients with spastic hemiparesis

**Subject**	**Gender**	**Stroketype**	**Lesion location**	**Paretic side**	**Dominance**	**F.M.**
P1	M	H	Right frontal-temporal	L	R	15
P2	M	H	Unavailable in medical records	R	R	18
P3	F	I	Left caudate nucleus and thalamus	R	R	19
P4	M	H	Left Internal Capsule	R	R	19
P5	M	I	Right cortical-subcortical precentral	L	R	21
P6	M	H	Unavailable in medical records	R	R	30
P7	M	I	Right cortical-subcortical parietal	L	R	36

The rightmost column reports the Fugl–Meyer clinical rating of loss of sensorimotor function in the arm: 15 = severe, 36 = minimal. Labels in the 3rd, 4th, 6th, and 7th columns refer to: *F* Female, *M* Male, *H* Hemorragic, *I* Ischemic, *L* Left, *R* Right.

### Experimental protocol

Each participant sat on a chair and gripped the handle of a planar manipulandum, the Inmotion2 Robot (Interactive Motion Technologies, Watertown, MA, USA) [30]. Trunk movements were prevented or minimized with a 5-point seatbelt. The right elbow was supported in the horizontal plane by a rigid support. The wrist was immobilized by a splint. Posture was adjusted depending on the subject’s body size and height. In particular, we properly adjusted the chair’s height to have the shoulder joint and the robot handle lying on the same plane, and the shoulder elevation-depression angle at about 90 degrees. Finally, subjects were positioned so that when in the central position, the angle between the arm and the forearm links was approximately 80–90 degrees.

### Experiments with healthy subjects

Experiments with healthy subjects aimed at understanding potential usage of the SVM classifier and quantifying its accuracy. These experiments allowed for the exploration of normal characteristics of EMG signals in terms of spatial distribution and the definition of a reference model of muscular activations.

**Figure 1:**
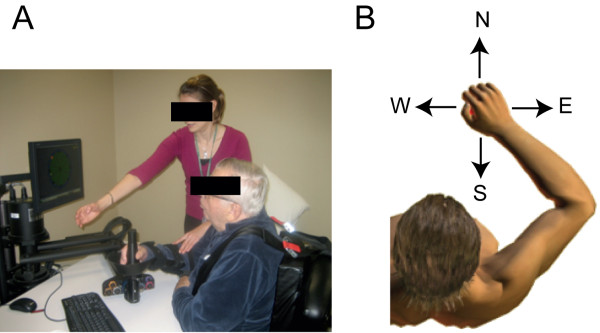
**The experimental setup. (A)** Robotic Therapy at Burke Rehabilitation Hospital. **(B)** Targets position distribution on the horizontal plane.

Participants were instructed to make point-to-point horizontal reaching movements between a central position and one of four outer targets arranged on a 0.14 m circumference at North, East, South, and West locations (see Figure 1). Each trial began once the robotic handle was positioned in the start position. After one second, subjects were prompted to initiate movement by an auditory cue. Subjects were instructed to perform the trial within a certain time frame and stop at target location for at least 1 s. Movements were performed for three different durations (1000, 600 and 300 ms). Visual and auditory feedbacks were provided when motion was outside the temporal constraints; unsuccessful movements were repeated. For each direction and each speed condition, subjects repeated the exercise 5 times for a total of 60 movements.

### Experiments with stroke subjects

Similar to typical robot-aided session therapy [30,32], subjects were required to reach out for 8 targets placed on the circumference of 0.14 m of diameter starting from a central position (Figure 1). No assistance was provided by the device throughout the experiment. No time constraints were applied to patients. They were instructed to stop in the target position. The exercise was repeated 5 times for each direction (total of 80 movements).

In all cases participants received a visual feedback of the target location and the movement of the robot handle by means of a computer monitor placed in front of them (Figure 1).

### Data acquisition and analysis

The optical encoders mounted on the handle of the Inmotion2 device enabled recording hand position with a frequency of 1000 Hz. In the case of GROUP I and patients #2 and #6, EMG signals of several muscles were recorded with active bipolar surface electrodes (Delsys, Boston, MA) at 1000 Hz. Each electrode was connected to an amplifier (total gain, 1000); signals were band-pass filtered (20–450 Hz) and then recorded by the robot (position and EMG synchronized). In the case of patients #1, #3, #4, #5 and #7, those who performed the experiments at the Volterra Hospital, EMG signals were recorded with the Noraxon data acquisition system (NORAXON, Telemyo 2400 T, V2). Sample rate was set at 1500 Hz and total gain was 1000. A synchronization signal was sent by the robot to the Noraxon system both when the cursor exited from the initial target and when it entered in the arrival target. This signal was used in post processing analysis to synchronize position and EMG data.

The EMG activities were recorded from a selection of muscles, thought to become active in similar exercises [12,33,34]: PM (Pectoralis Major), DP (Deltoid Posterior), DM (Deltoid Medial), DA (Deltoid Anterior), BI (Biceps Brachii), TR (Triceps Brachii, lateral head), UT (Trapezius Superior), MT (Trapezius Medial), LT (Latissimus Dorsi), TM (Teres Major). In the case of GROUP I we could record up to 8 external channels, thus the protocol was repeated twice (muscles recorded in the first block: PM, DP, DM, DA, BI, TR, UT; muscles recorded in the second block: DP, DM, BI, TR, UT, MT, LT, TM). Proper electrode placement was verified for each muscle by asking subjects to perform both free movements and isometric contraction (when possible) according to testing procedures specified in [35] and then observing the expected activation patterns. Only motions toward North, East, South, and West directions were analyzed. The position data were low-pass filtered (Butterworth filter; 15 Hz cutoff; Matlab filtfilt function) and differentiated to compute tangential velocity. Movement onset and completion were determined via a 5% of peak speed threshold. When acquired with the Noraxon system, EMG signals were first bandpass filtered (20–450 Hz). In all cases, they were high-pass (Butterworth 10 Hz cut-off filter) and low-pass filtered (moving average 20 ms).

In the case of Patients #1, #4 and #7, MT muscle in some cases showed a change in signal amplitude during the experiment, likely resulting from a partial detachment of the electrode from the skin. This muscle was then removed from further analysis.

### Classification and validation procedure using support vector machine (SVM)

Initial intended movement direction was extrapolated from muscle activity recorded between [−100; 100] ms with respect to the movement onset, represented by the time the cursor left the start target. Due to the stochastic nature of the EMG signal, we evaluated the discrimination capability and robustness of several features used in the literature for myo-control classification (for a detailed review see [19]). Time domain features were preferred to more complex time frequency representations because of the lower computation cost and the good classification performance [29,34,36]. In particular, we tested the following EMG parameters:

•IAV (Integral of Absolute Value): $\mathrm{IAV}=\frac{1}{N}\sum_{i=1}^{N}\left|x_{i}\right|$, which estimate the integrated absolute value of the signal in a segment length of N samples.

•AR coefficients (Auto-Regressive coefficients): time series in which the signal samples are estimated by linear combination of their earlier samples [19]. The order of the AR model was set at 4 in accordance with previous studies [34].

•ZC (Zero Crossing parameter): the number of times the waveform crosses the zero:

•

$$\begin{array}{l} \mathrm{ZC}=\sum_{i=0}^{N}\mathrm{sign}\left(-x_{i}*x_{i+1}\right)\;\mathrm{where}\;\mathrm{sign}\left(x\right)=\left\{\begin{array}{l} 1\;\mathrm{if}\;x>\mathrm{threshold}\\ 0\;\mathrm{otherwise} \end{array}\right) \end{array}$$

Threshold value was set as 0.025 V.

•HIST (HISTogram parameter) [37]. For each muscle, the maximal activation value across conditions and repetitions was first computed and then used to define a muscle specific reference voltage range, symmetric with respect to the baseline. This range was subdivided in bins, and the frequency (i.e., the histogram) with which the EMG recording falls within each of the voltage bins, was computed. Similar to [37] the number of bins was set to 9.

The quality of the feature space was estimated by means of the Davies Bouldin (DB) parameter applied to the scatter matrix of data from all muscles as in [38]. The DB index estimated how significantly a cluster overlaps its neighbors. It was obtained through averaging the worst case separation of each cluster from the others based on:

(1) $$\mathrm{DB}=\frac{1}{K}\sum_{i=1}^{K}\max\left(R_{\mathrm{ij}}\right)\;\mathrm{with}\;i\;\neq \;j$$

Where,

(2) $$R_{\mathrm{ij}}=\frac{S_{i}+S_{j}}{D_{\mathrm{ij}}}$$

where S_i_ and S_j_ are the dispersion of the i_th_ and j_th_ clusters respectively

(3) $$S=\sqrt{\left\{\frac{1}{N}\right\}\sum_{j=1}^{N_{i}}{\left(y_{i}-m_{i}\right)}^{T}\left(y_{j}-m_{1}\right)}$$

and D_ij_ is the distance between mean values:

(4) $$D_{\mathrm{ij}}={\left\{{\left(m_{i}-m_{j}\right)}^{T}\left(m_{i}-m_{j}\right)\right\}}^{1/2}$$

with N_i_ is the number of cluster C_i_, y_i_ is the i^th^ input pattern vector and m_i_ is the mean vector of pattern i^th^.

The lower the DB parameter, the higher is the degree of class separability. The comparison among all subjects and movement directions showed that HIST parameter presented the best overall performances--see Table 2.

**Table 2:** Application of the Davies–Boulding in method for the signal features selection

**Parameter**	**DB**
HISTogram parameter ( HIST )	0.53
Zero Crossing parameter (ZC)	0.70
Auto Regressive coefficients (AR)	1.01
Integral Absolute Value (IAV)	1.24

The HIST parameters were clustered using the Support Vector Machine algorithm (LIBSVM library, http://www.csie.ntu.edu.tw/~cjlin/libsvm[39], Gaussian radial basis function, sigma = 2), a supervised learning method used for classification [40]. Formally, the SVM constructs a set of hyper planes in the dimensional space that separate between a set of objects having different class memberships. In the present application, the classes are represented by the four movement directions, and the objects are the 9-components HIST vectors parameters. Accurate classification is achieved when the hyper planes present the largest separation.

Different validation tests were then carried out to assess the accuracy of SVM as described in next sections.

#### Validation of SVM with GROUP I

Principal component analysis (PCA) was carried out using data from healthy subjects in order to decrease the number of muscles relevant to motion. This approach aimed at building a low dimensional feature space and reducing computation resources demand in terms of time and memory of the classifier. We ended up with 7 of the 10 muscles recorded during the experiment (PM, TR, DA, DP, DM, BI, UT). Therefore, each movement was described by a vector of 63 components (9 values × 7 muscles).

For healthy subjects the classifier has been:

1. Trained (70% of the trials) and tested (30% of the trials) individually, i.e., with the data of each subject (TEST 1). This test evaluated the performance of the classifier when calibrating the system on each subject. Misclassification would then be ascribed to intra-subject’s variability.

2. Trained (70% of the total amount of data) and tested (30% of the total amount of data) with the composite of all subjects’ data, i.e., with the data of all the 9 subjects enrolled in the study (TEST 2). This test aimed at evaluating the possibility of building a general model of muscle patterns activation for GROUP I. A higher correct classification rate would imply a lower inter-individual variability.

3. Trained with a composite of 7 subjects and validated with the 2 remaining subjects selected as the 2 worst (i.e., the subjects who showed lower success rates in TEST 1), or the 2 best (i.e., the subjects who showed higher percentage of correct classification in TEST 1). Those tests aimed at characterizing the classifier performance depending on the quality of training and testing data.

In all cases, training and validation data were randomly selected and were mutually exclusive. TESTS 1 and 2 were run 20 times each: success rate was computed as the average of the percentages of correct classification observed in each iteration.

### Validation with GROUP II

Different tests were carried out to evaluate the classification accuracy of the SVM in the case of stroke patients. In particular, SVM was:

1. Trained (70% of the trials) and tested (30% of the trials) individually, using the muscle selection extrapolated for healthy subjects (TEST 1). This test aimed at the characterization of intra-subject’s variability within pathological subjects.

2. Trained with data recorded for GROUP I and tested individually on each patient (TEST 2). This test aimed at the evaluation of misclassification in relation to the presence of abnormal patterns of muscle activation.

3. Trained (70% of the trials) and tested (30% of the trials) individually, including all recorded muscles (TEST 3). It aimed at exploring whether the inclusion of muscles acting on the trunk and on the shoulder could improve the accuracy of the classifier. In other words, we investigated the possibility of the presence of compensatory strategies adopted to restore the functionality of the arm.

In the case of TEST 1 and TEST 3, training and test samples were randomly selected among the dataset. Each test was run 5 times, and the success rates were averaged to evaluate the total accuracy.

### EMG spatial distribution index for classification and assessment

To investigate abnormal patterns of muscle activation at the base of possible misclassification, we exploited a graphical approach to represent EMG spatial characteristics distribution. PCA analysis was computed for each movement and each subject and applied to the covariance matrix of the EMG raw signals within [−100; 100] ms with respect to the movement onset. The relevance of each muscle in the specific motion direction under investigation for each trial was quantified by means of an information content index, namely the Coefficient of Expressiveness (CoE) [34].

For each *j*^*th*^ trial, each *l*^*th*^ muscle, and each *r*^*th*^ direction, we computed a *k* index defined as:

(5) $$\begin{array}{l} k_{j,l,r}=\sum_{i=1}^{n}\left|c_{i,l,r}\right|\frac{\lambda_{i}}{\sum_{m=1}^{p}\lambda_{m}}\;j=\;1,\;J \end{array}$$

where n is the number of principal components that contains 80% of the variance of the system, c_i,l,r_ is the correlation coefficient between the i^th^ principal component and the selected muscle for the r^th^ movement direction, λ_i_ is the eigenvalue associated to the i^th^ principal component, p is the total number of eigenvalue, J is the numbers of trials. The CoE coefficient for each muscle was given normalizing the k_j,l,r_ index with respect to the maximum among the k indexes of the muscles recorded for the j^th^ trial.

Muscle activity during each trial was then summarized via N-component vector of CoE values, where N was the number of the muscles included in the analysis. Only muscles with CoE values larger than 0.7 were considered relevant for motion production; otherwise they were judged non-relevant (i.e., CoE = 0).

For each muscle, the modulation of the CoE across the four directions was evaluated considering the polar distribution of the coefficient. Specifically the patterns of muscle activations identified for the neurological intact participants were used as a baseline for comparison with those of the patients. Thus, different abnormal spatial patterns were evaluated and correlated with the results obtained with the SVM.

## Results

### SVM prediction: GROUP I

Table 3 summarizes the results of the different validation tests carried out for GROUP I. As expected, training the SVM individually led to a high rate of success (93.9 ± 4.4%), indicating a lower incidence of intra-subject’s variability on the classifier accuracy. It remained high at 89.6 ± 4.4% when training was done with the composite of data of all the subjects enrolled in the experiment. Table 4 shows the “confusion matrix,” indicating the frequency of correct and misclassified directions in the validation TEST 2. Results showed that the highest number of misclassification was observed between North and East directions. When validating the classifier with the two subjects characterized by the worst performance in the individual training (TEST 3), the rate of success dropped to a mean value of 79.1%. Confusion matrix indicates that the worst recognized direction was North, classified in 55% of the cases as East; also, in 14.3% of cases South direction was classified as North, probably due to co-contraction of the antagonist muscles. Finally, validation carried out with data from the subject who showed the higher success rate during the individual tests returned the highest classification accuracy (97.5%).

**Table 3:** GROUP I: classification results

**Test type**	**Classification**
	**Results (%)**
TEST 1 SVM trained and tested with individual data	93.9 ± 4.4
TEST 2 SVM trained with dataset from a composite of all subjects and tested with the remaining dataset	89.6 ± 4.4
TEST 3 SVM trained with dataset from a composite of 7 subjects and tested with a dataset from 2 worst subjects	79.1
TEST 4 SVM trained with dataset from a composite of 7 subjects and tested with a dataset from 2 good subjects	97.5

Percentage of correct classification for each test carried out in the case of healthy subjects.

**Table 4:** **GROUP I: classification results of TEST 2-3-4 (see** Methods)

**Test type**	**Actual classes**	**Predicted classes**			
		**North**	**East**	**South**	**West**
TEST 2	North	84.1	11.7	5.6	0
	East	12.1	88.3	0	0
	South	3.8	0	91.8	6.6
	West	0	0	2.6	93.4
TEST 3	North	45	0	14.3	0
	East	55	100	0	0
	South	0	0	71.4	0
	West	0	0	14.3	100
TEST 4	North	100	10	0	0
	East	0	90	0	0
	South	0	0	100	0
	West	0	0	0	100

### SVM prediction: GROUP II

Tables 5, 6, and 7 show the classification results and the confusion matrices for all the tests carried out to evaluate the classifier performance for each pathological subject. Compared to healthy subjects, the classification rates dropped dramatically. Misclassifications were present in all directions and were differently distributed across patients.

**Table 5:** GROUP II: classification results and confusion matrices for TEST2

**Patient id and classification rate**	**Actual classes**	**Predicted classes**			
		**North**	**East**	**South**	**West**
P1	North	40	0	0	40
35%	East	0	0	0	0
	South	60	100	100	60
	West	0	0	0	0
P2	North	20	0	0	0
35%	East	60	20	0	0
	South	20	80	100	100
	West	0	0	0	0
P3	North	80	100	80	100
25%	East	20	0	0	0
	South	0	0	20	0
	West	0	0	0	0
P4	North	20	0	0	0
30%	East	0	0	0	0
	South	80	100	100	100
	West	0	0	0	0
P5	North	40	0	0	0
45%	East	60	40	0	40
	South	0	60	100	60
	West	0	0	0	0
P6	North	20	0	0	0
35%	East	0	0	0	0
	South	80	100	100	100
	West	0	0	0	0
P7	North	0	0	20	0
35%	East	20	0	0	0
	South	80	80	80	40
	West	0	20	0	60

Elements on the left-right diagonal indicate the percentage of correct classification, while elements on the off-diagonals denote percentage of misclassification. SVM was trained with data from GROUP I and validated individually on each patient with the muscle dataset selected for healthy subjects (see Methods).

**Table 6:** GROUP II: classification results and confusion matrices for TEST1

**Patient id and classification rate**	**Actual classes**	**Predicted classes**			
		**North**	**East**	**South**	**West**
P1	North	28.6	25	0	58.3
43.3%	East	14.3	75	28.6	16.7
	South	42.9	0	71.4	0
	West	14.3	0	0	25
P2	North	100	0	0	0
70%	East	0	77.8	0	0
	South	0	22.2	85.7	60
	West	0	0	14.3	40
P3	North	42.9	26.6	0	0
30%	East	0	28.6	66.7	28.6
	South	28.6	42.9	11.1	28.6
	West	28.6	0	22.2	42.9
P4	North	12.5	57.1	0	0
26.7%	East	87.5	28.6	0	0
	South	0	14.3	37.5	71.4
	West	0	0	62.5	28.6
P5	North	14.3	0	0	20
40%	East	28.6	57.1	16.7	20
	South	14.3	42.9	83.3	40
	West	42.9	0	0	20
P6	North	83.3	0	0	0
70%	East	16.7	45.5	12.5	0
	South	0	54.5	75	0
	West	0	0	12.5	100
P7	North	100	0	0	0
66.7%	East	0	57.1	60	0
	South	0	42.9	40	0
	West	0	0	0	100

Elements on the left-right diagonal indicate the percentage of correct classification, while elements on the off-diagonals denote percentage of misclassification. SVM was trained (70% of trials) and tested (30% of trials) individually on each patient with the muscle dataset selected for healthy subjects; training and testing data were randomly selected and mutually exclusive; test was repeated 5 times for each patient and accuracy rates were averaged across iterations.

**Table 7:** GROUP II: classification results and confusion matrices for TEST3

**Patient id and classification rate**	**Actual classes**	**Predicted classes**			
		**North**	**East**	**South**	**West**
P1	North	66.7	20	25	14.3
53.3%	East	16.7	40	41.7	0
	South	0	20	33.3	0
	West	16.7	20	0	85.7
P2	North	100	0	0	0
70%	East	0	87.5	0	0
	South	0	0	27.3	0
	West	0	12.5	72.7	100
P3	North	66.7	25	0	0
36.7%	East	16.7	75	75	50
	South	0	0	0	0
	West	16.7	0	25	50
P4	North	28.6	12.5	0	12.5
36.7%	East	57.1	37.5	14.3	12.5
	South	14.3	50	57.1	50
	West	0	0	28.6	25
P5	North	25	14.3	0	33.3
43.3%	East	37.5	57.1	16.7	0
	South	12.5	28.6	83.3	44.4
	West	25	0	0	22.2
P6	North	87.5	0	0	0
83.3%	East	12.5	71.4	12.5	0
	South	0	28.6	75	0
	West	0	0	12.5	100
P7	North	100	0	0	14.3
70%	East	0	50	55.6	14.3
	South	0	50	44	0
	West	0	0	0	71.4

Elements on the left-right diagonal indicate the percentage of correct classification, while elements on the off-diagonals denote percentage of misclassification. SVM was trained (70% of trials) and tested (30% of trials) individually on each patient with the muscle dataset recorded during the experimental session (see Methods); training and testing data were randomly selected and mutually exclusive; test was repeated 5 times for each patient and accuracy rates were averaged across iterations.

When SVM was trained with data from the healthy subjects and tested separately on each patient, the accuracy rates ranged between 25-45% (Table 5). To give an overview of the classifier performance, in the following we reported the classification results observed in each direction obtained pulling together the data from all pathological subjects. Motion toward the North direction was correctly detected in 31.4% of the trials (misclassification rates: East - 22.9%, South - 45.5%). Poor classifier performances were observed when aiming to move toward the East direction, correctly recognized in only 8.6% of the cases (misclassification rates: North - 14.3%, South - 74.3% , West - 2.8% ). South direction was detected correctly 85.7% of the time (misclassification rates: North - 14.3%). Finally, motion toward the West direction showed a success rate of 8.6% (misclassification rates: North - 20% -East - 5.7%, South 65.7%).

Training and testing the SVM individually (TEST 1 and TEST 3, Tables 6 and 7) resulted in a higher percentage of correct classification than training SVM with the model extracted from healthy subjects. When using the seven-muscles subset extracted for healthy subjects (i.e., TEST 1), classification rates ranged between 30-70% (Table 6). Motion intended direction was correctly detected in 54.5% of total trials toward the North (misclassification rates: East - 21% , South - 12.3% West - 12.2% ), in 52.8% of the total trials toward the East (misclassification rates: North - 15.7%, South - 31.5%), in 57.7% of the total trials toward the South (misclassification rates: East - 26.4%, West - 15.9%), and in 50.9% of the total trials toward the West direction (misclassification rates: North - 11.2%, East - 9.3%, South - 28.6).

When trained and tested on individual data including all the recorded muscles (i.e., TEST 3, Table 7), accuracy rates were slightly higher and ranged between 36.7-83.3%. When aiming toward the North direction, the accuracy rate was 67.8% (misclassification rates: East - 20.1%, South - 3.8%, West - 8.3%). When moving toward the East direction, the correct classification rate was 59.8% (misclassification rates: North - 10.3%, South - 25.3%, West - 4.6%). Finally, motion toward South and West directions was detected correctly in 45.8% (misclassification rates: North - 3.6%, East - 30.8%, West - 19.8%), and 64.9% (misclassification rates: North - 10.6%, East - 11%, South - 13.5%) of the cases, respectively.

### EMG spatial distribution

**Figure 2:**
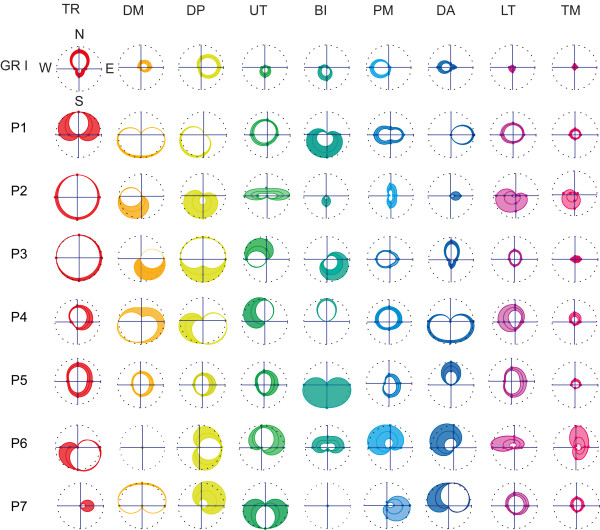
**CoE parameter directional tuning.** Modulation of the CoE parameter across the four aimed directions (N, E, S, W) for each recorded muscle are shown respectively for the healthy subjects group (GR I) and for each patient enrolled in the study. CoE coefficients of each muscle were averaged across repeated trials and, in the case of GR I, also across different subjects (CoE_M). The polar diagrams show the distribution of the CoE_M ±1.96 · SE (SE = Standard Error) coefficients connected by periodic cubic spline interpolation curves. The resulting area inside the curves represents the 95% confidence interval of the CoE distribution for each muscle. Dashed black lines are circles of unit radius. Muscles were ordered according to their relevance in motion production in each direction observed for GR I: in particular TR was responsible for motion toward North direction, DM and DP toward East direction, UT and BI toward South direction, and PM and DA toward West direction.

Spatial characteristics of the muscle EMG activation relative to the initial part of the movement were evaluated considering the polar distribution of the CoE coefficients, which were averaged across repeated trials and different subjects (CoE_M). The CoE_M ±1.96 · SE (SE = Standard Error) coefficients computed for each direction were connected by periodic cubic spline interpolation curves (Figure 2). The resulting area inside the curves represented the 95% confidence interval of the CoE distribution. The analysis of the spatial distribution of the CoE parameter was used to explain and interpret the observed misclassification rates of the classifier.

### Healthy subjects

**Figure 3:**
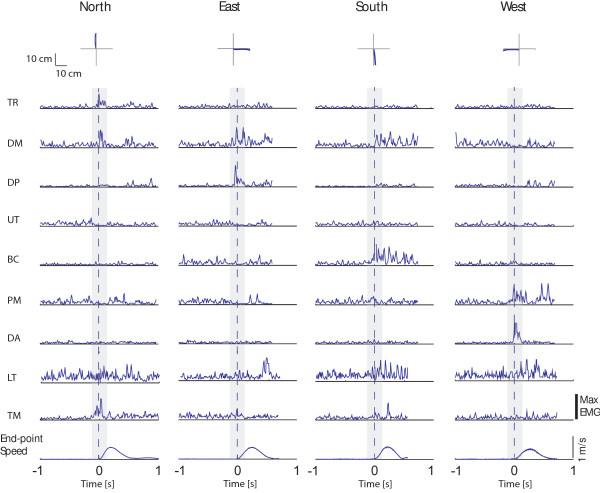
**Examples of end-point kinematic and EMG signals collected during one trial in the 600 ms condition from one healthy subject.** Top panel: endpoint trajectories are shown for each movement direction (columns). Central panels: EMG signals are shown for each muscle (rows) and movement direction (columns); data were full wave rectified and normalized with respect to the maximum of the specific muscle over all conditions, filtered (see Methods section), and integrated over 10 ms intervals; the gray area represents the time window used for the present analysis; muscles abbreviation are defined in the Methods section. Bottom panel: tangential velocity profiles are shown for each movement direction (columns). Data are aligned to the movement onset.

In Figure 2, muscles were ordered according to their relevance in motion production observed for healthy subjects from the analysis of the EMGs raw data (i.e., Figure 3). The TR muscle showed a higher CoE value when moving toward the North direction; similarly, DM and DP contributed more when aiming toward the East direction, although often active also when moving toward the North direction; UT and BI presented a larger activity when aiming toward the South direction and PM and DA toward the West direction (Figure 2, *first row*). This approach took into consideration modulation of muscle activity reported by Flanders and Georgopoulus [22,23]. In accordance with those studies, we observed that muscles relevant when initiating motion toward one direction, i.e., playing an agonist role, were not activated in the opposite direction where they acted as antagonist. Some episodic, abnormal co-activational patterns at the base of SVM misclassification were observed. They were presumably related to the need for mechanical stabilization of the arm, especially in the case of a fast movement condition (movement time 300 ms). For example, the activation of the elbow flexor (BI) sometimes required the activation of the shoulder adductor (PM) to stabilize the shoulder. Similar relationships were present between elbow extensor (TR) and shoulder abductors (DP).

Overall, GROUP I showed highly repeatable and stable patterns of activation across different subjects, trial repetitions and movement velocities as revealed by the narrow confidence interval of the CoE parameter. Conspicuously, in accordance with PCA analysis, muscles of the back--such as MT, LT, TM--were not relevant for motion production as indicated by the lower CoE values.

### Stroke subjects

**Figure 4:**
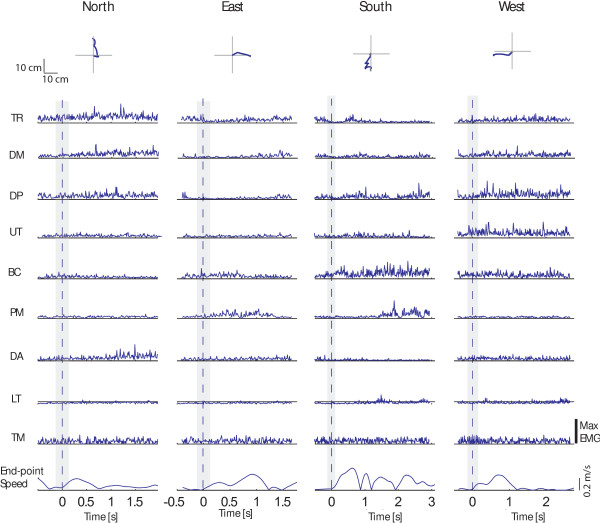
**Examples of end-point kinematic and EMG signals collected during one trial from one pathological subject (P1).** Top panel: endpoint trajectories are shown for each movement direction (columns). Central panels: EMG signals are shown for each muscle (rows) and movement direction (columns); data were full wave rectified and normalized with respect to the maximum of the specific muscle over all conditions, filtered (see Methods section), and integrated over 10 ms intervals; the gray area represents the time window used for the present analysis; muscles abbreviation are defined in the Methods section. Bottom panel: tangential velocity profiles are shown for each movement direction (columns). Data are aligned to the movement onset.

In accordance with previous studies [41] we observed that EMG signals were quite different from those observed in healthy subjects in almost all the pathological subjects enrolled in the study (for example, see Figures 3 and 4). A graphical overview of muscle patterns characteristics relative to the initial phase of the movement, described by means of the CoE parameter distribution, was reported for each subject in the polar plots of Figure 2.

In the case of Patient #1, TR contributed to motion toward North, West and East directions; DM and the DP were involved mainly when initiating motion toward the South and West directions and the DA muscle when moving toward the East direction (see Figures 2 and 4). Patient #2 presented no specific directional tuning of the CoE parameter relative to the TR muscle; the UT muscle was involved more when aiming toward the lateral directions (i.e., the West and East directions) than toward the South direction, as in the case of healthy subjects; the PM muscle showed the higher CoE values when moving toward back and forward directions (i.e., North and South directions). Patient #3 showed no specific directional tuning of the CoE parameter of both the TR and DP muscles; the UT muscle was mainly involved when the subject extended the forearm toward the North and the West directions; the DA muscle was recruited when moving toward the North direction. Patient #4 showed co-activational patterns of agonist and antagonist muscles: notably the DP and the DA muscles when moving toward the West direction, and the TR and the BI muscles moving toward the North direction. Patient #5 showed no specific directional tuning of the CoE parameters of several muscles--i.e., the DM, DP, UT, PM and LT muscles--while the DA muscle often contributed to initiate motion toward the North direction. Patient #6 presented abnormal co-activational patterns between elbow extensor and flexor muscles: the CoE parameters relative to the activity of the TR muscle were higher when initiating the movement toward the South direction, while those relative to the BI muscle were higher when moving toward the North direction. Finally, in the case of Patient #7, the TR and PM muscles contributed mainly to motion toward the East direction, and the BI muscle was not relevant in any direction as showed by the null CoE coefficients.

In almost all patients, there was a larger involvement of the back muscles in motion production with respect to GROUP I, as shown by the higher CoE coefficient of the LT and TM muscles reported in Figure 2.

Summarizing, the analysis of the CoE parameter distribution in the case of pathological subjects showed that abnormalities varied idiosyncratically and were ascribed to several factors, such as: a shift of the preferred muscle activation direction, co-contraction of antagonist muscles, and the presence of abnormal coactivational patterns.

## Discussion

Therapeutic robotics started about 20 years ago. Because robots can be used to reproduce different multi-sensory interactive scenarios, applying robot-assisted therapy allows us to customize the interventions on individual physical impairments [9,30,42,43], and to also provide precise control over a large number of physical variables--haptic, visual, and auditory cues--that influence motor behavior [10,32]. Here we explored the efficacy of using EMG signals, not as a measure of the strength of specific groups of muscles [12,42] but as a way to detect intentions to move toward a certain direction. This classification could be used in future applications to enable the control of assistive and rehabilitation robotic devices. With this aim in mind, we examined whether we could build a static functional map of the EMG activation patterns for point-to-point reaching movements located on the horizontal plane environment. We used the SVM algorithm to predict the intended motion direction with the highest possible accuracy and to understand whether and how the inter- and intra-subjects’ variability could affect repeatability of the measurements.

### Limits of EMG pattern recognition

The approach was quite successful for healthy subjects. In this case, we were able to achieve a classification accuracy of 89.6 ± 4.4% (TEST 2, Table 2). Even more interesting, we employed the data from all subjects and did not train the algorithm on individual data. Inter- and intra-subject’s variability was not a critical factor affecting the classifier performance, as shown by the higher classification rates obtained with TESTS 2, 3 and 4 reported in Table 2. Accuracy increased significantly, i.e., up to almost 97.5% of the success rate when the algorithm was trained and tested on individual data or when validating the SVM with our best subjects. However, we were not able to obtain 100% correct predictions in any case. While the accuracy could be increased further (for instance with the use of more sophisticated techniques [19,36,44]), the results obtained in the present analysis are in line with different techniques used in the past for similar applications. For example, it has been reported an accuracy close to 100% using a neuro-fuzzy classifier to detect planar arm movements similar to those tested in the present study [34]. In the case of EMG based control of prosthetic devices, several studies reported a success rate ranging between 94-99% [17,45,46]. Other classification algorithms, such as the Hidden Markov Models (HHM) and Higher Order Statistics (HSO), have also been tested but the results have not been very promising so far [47,48]. Finally, brain computer interfaces (BCI) used to command computer devices with upper extremity muscle activity achieved a remarkable 96-97% recognition of individual intentions [16]. Of note, in all the cited studies the classifier was tuned to the specific individual, as for TEST 1 in the present analysis.

Overall the present analysis showed that in the case of healthy subjects, beside the presence of episodic abnormal co-activational patterns at the base of SVM misclassification, EMGs can provide a reliable map of the coactivation relationships between groups of muscles.

### EMG pattern recognition in robot-mediated neuro-rehabilitation

In the case of stroke patients, the classifier accuracy dropped dramatically and ranged between 25% and 45% (Table 5). Mainly, the classifier failed to recognize motion toward the East and the West directions (i.e., the average accuracy rates were 8.6% in both cases). Consistent differences in the EMG patterns between the two groups were also observed (see Figures 2, 3, and 4). The analysis of the CoE directional distribution revealed the presence of individual abnormal patterns of activation--spastic muscular restraint, muscle synergies, lack of elbow extension, weakness of specific muscle coordination deficits--in accordance with previous studies [8,12,26,27,33,41,48-52]. For instance, the simultaneous activation of the shoulder abduction and elbow flexion, i.e., flexor synergy [41,53,54], was present in several subjects (Patient #1, #2, #3, #4 and #5). Both DM and DP muscles were no longer activated with the elbow extensor and the TR muscle, and showed a high CoE coefficient when attempting to move toward the South direction. This could explain the misclassification rates observed when attempting to move toward the North (i.e., 68.6%) and East directions (91.4%), as well as the large amount of trials misclassified in the South direction (Table 5).

The number of correct classifications increased when the system was trained and tested on individual data (Tables 6 and 7). The best classifier’s performances were achieved when a larger number of muscles was used, i.e., including the LT and TM muscles (Table 7), in accordance with previous studies which reported an increase in the involvement of the trunk in post stroke upper limb movement coordination [53]. With this approach it was possible to achieve up to 83.3% of correct classification, as in the case of Patient #6. Nevertheless, for the other patients the accuracy was not as high as in the case of GROUP I and ranged between 36.7 -70%. In this context, it is important to note that an accuracy of 36.7% was close to the probability of getting a particular direction by chance alone, which in the present case was 25% given that the classifier had to discern between four very distinct possible classes. Overall the accuracy in the East and West directions increased up to 59.8% and 64.9%, respectively. To achieve a better classification performance, SVM generated the hyper planes that increase the separation between the different classes. The solutions exploited varied according to the subject specific EMG signal characteristics, that is, the distribution of the HIST vectors in the task space. One of the possible drawbacks of this strategy was that the system might have recognized the intention to move toward a certain direction from the pathological incorrect schemes or the stereotypical coupling muscle patterns. Moreover, the method was affected by the presence of large variability of the EMG features within each class. In fact, each muscle presented activation over a broader range of directions compared to healthy subject groups as shown by the large confident intervals of the CoE parameter distribution in Figure 2. It has been largely documented that stroke patients present an increased movement variability with respect to normal population [49,53,55]. The end point kinematic is often characterized by large path errors and a speed profile composed of several low amplitude peaks, markers of discrete sub-movements that underlie deficits in motor control [33,41,54-57]. We also observed these characteristics in the performance of the stroke patients enrolled in this study (Figure 4). In this context, compensatory strategies and corrective actions exploited by patients in their attempt to complete the task might have been changed from trial to trial as also reported in previous studies [53].

Summing-up, the approach was not successful in the case of pathological subjects. The aimed direction was not predictable with a sufficient accuracy whether using the general map extracted from data of normal subjects (i.e., reference model), or tuning the classifier on each individual.

Some limitations of the study should be highlighted at this point. For instance, we only recorded a small subset of all the possible trunk and upper limb muscles. In the case of GROUP I, this approach worked quite well. However, in the case of GROUP II the number of trunk and back muscles should have been increased to account for the presence of compensatory strategies in the classifications extracted with the SVM. Proper electrodes placement was a critical issue when dealing with patients due to the weak EMG signals. Moreover, patients were often overweight due to physical inactivity with a subsequent EMG signal decay, which further affected classification accuracy. Additionally, we could not rule out the presence of fatigue at the base of intra-subject variability in the performance of stroke survivors. Finally, in the present study we used a statistical classifier. Neural networks might also have been employed as an alternative, given the good performances in the pattern recognition described in literature [19,34]. A recent study [36] however, reported no significant differences in the classification between the two approaches.

### New therapies and solutions

The results of this study are in line with a previous work from Lee and colleagues [44], which applied a subject-specific EMG patterns classification technique to discern the intent of stroke patient in performing six different manual tasks. To this aim they used the LDA (Linear Discriminant Analysis) classifier on segments of data of 150 ms in duration shifted in 100 ms increments. While a similar approach could be applied also to the present analysis to improve recognition accuracy, classification performances were nonetheless comparable to those herein reported. Specifically, mean recognition accuracy was 71.3% for moderately impaired subjects and 37.9% for severely impaired stroke. The poor classifier performances observed in the case of the severely impaired patient group, posits an important issue in our view. In fact, there might be a problem when using the classification to enable volitional control of assistive devices. For instance, if the patient produces uncoordinated activation patterns, which are not correctly interpreted by the classifier system, the robot could move in an undesired way. Moreover, having the system calibrated on the data from each patient might be not practical in clinical application. It took us over 3 hours to collect a patient’s data with the support of a research engineer and a research therapist. While it is possible to automate the process to collect the data by a regular clinician within an hour, it will require significant design effort and, in lieu of our poor results classifying 4 very distinct classes in severe stroke, lead to a low cost/benefit. Finally, there is the theoretical possibility of “bad” plasticity: the classifier could recognize the intended motion direction while reinforcing some unwanted pathological incorrect schemes [58,59].

It is often speculated that the process of motor recovery either involves spared tissue on the motor and sensory areas in the lesioned hemisphere or it enhances activity in pre-existing motor networks in the unaffected hemisphere. Several solutions employing EMG are possible. For example, it could be possible to calibrate the system on patient-specific characteristics and to have the clinician select the admissible co-activation pattern that represents an effective strategy to inhibit pathological scheme. The control system of the robot could then be implemented in order to train patients to always reproduce the same correct pattern. The rationale underlying this approach is that learning may be elicited providing the subject a feedback on the muscle forces he has to produce to achieve the desired trajectory.

## Conclusions

The aim of the present study was to investigate the use of EMG pattern recognition approaches, based on statistical classifiers, to decode subject’s intention to move toward a certain direction in the horizontal plane. In the case of normal subjects the approach worked quite well. In the case of stroke patients the approach did not perform well. Our results show the limitation of the use of this technique in robotic-aided neuro-rehabilitation; the findings suggest that rather than using the EMG signals to discriminate patient’s intentions, we could instead use these signals to develop an online procedure that provides a feedback on the error in the muscle activational patterns. Further experiments need to be performed to verify the clinical advantages which can be achieved using this approach.

## Competing interests

H. I. Krebs is a co-inventor in several MIT-held patents for the robotic technology. He holds equity positions in Interactive Motion Technologies, Watertown, MA, USA, the company that manufactures this type of technology under license to MIT.The other authors do not have competing interests, as defined by the BioMed Central Publishing Group, or other interests that may influence the results and discussion reported in this study.

## Authors’ contributions

BC conceived and designed the study, carried out the experiments and the data analysis and drafted the manuscript; PT carried out the experiments with patients; SM and HIK participated in the design and the coordination of the study and drafted the manuscript. All authors read and approved the final manuscript.
